# In-Feed vs. In-Water Chlortetracycline Administration on the Fecal Prevalence of Virulence Genes and Pathotypes of *Escherichia coli* Involved in Enteric Colibacillosis in Piglets

**DOI:** 10.3390/microorganisms13061185

**Published:** 2025-05-22

**Authors:** Ramya Kalam, Raghavendra G. Amachawadi, Xiaorong Shi, Jianfa Bai, Mina Abbasi, Mike D. Tokach, Tiruvoor G. Nagaraja

**Affiliations:** 1 Department of Clinical Sciences, College of Veterinary Medicine, Kansas State University, Manhattan, KS 66506, USA; rkalam@vet.ksu.edu; 2 Department of Diagnostic Medicine/Pathobiology, College of Veterinary Medicine, Kansas State University, Manhattan, KS 66506, USA; xshi@vet.ksu.edu (X.S.); minaabbasi@vet.ksu.edu (M.A.); tnagaraj@vet.ksu.edu (T.G.N.); 3 Kansas State Veterinary Diagnostic Laboratory, College of Veterinary Medicine, Kansas State University, Manhattan, KS 66506, USA; jbai@vet.ksu.edu; 4 Department of Animal Sciences and Industry, College of Agriculture, Kansas State University, Manhattan, KS 66506, USA; mtokach@ksu.edu

**Keywords:** colibacillosis, chlortetracycline, *Escherichia coli*, pathotypes, PCR, piglets, virulence genes

## Abstract

Colibacillosis in nursery pigs, caused by *Escherichia coli* (ETEC, EPEC, and STEC pathotypes), remains a major economic concern in the swine industry. This study evaluated the effects of in-feed or in-water chlortetracycline (CTC) administration on the fecal prevalence of virulence genes and pathotypes associated with colibacillosis. A total of 1296 weaned piglets (21 days old) were allocated to 48 pens (16 pens/treatment; 27 piglets/pen) and assigned randomly to no CTC, in-feed CTC, or in-water CTC groups. CTC was administered from days 0 to 14. Fecal samples from five piglets per pen on days 0, 14, and 28 were enriched, screened by 11-plex PCR, cultured for pathotypes, and tested for CTC susceptibility and tetracycline resistance genes. None of the 360 fecal samples or 3267 *E. coli* isolates were positive for *bfpA* or *aggA*. Prevalence of *estB* (96.9%) and *astA* (92.8%) was highest. ETEC was the dominant pathotype (41.2%), with *astA* (29%) and *estB* (21.9%) as predominant enterotoxin genes. CTC administration had no significant effect on fecal prevalence of virulence genes or pathotypes (*p* > 0.05). *stx2* and STEC were detected only at day 28, all harboring *stx2e*. All pathotypes were CTC-resistant, with *tetA* as the predominant resistance gene.

## 1. Introduction

Enteric infections caused by *Escherichia coli* in swine, particularly in nursery pigs, are of significant economic importance in the swine industry [1]. Three diseases, neonatal enteritis, post-weaning diarrhea, and edema disease, collectively called ‘colibacillosis’, caused by different pathotypes of *E. coli* are common in neonatal and weaned piglets. The economic impacts of colibacillosis are because of mortality, morbidity, and costs associated with treatment and vaccinations [2,3]. The pathotypes involved in colibacillosis are enterotoxigenic (ETEC), enteropathogenic (EPEC), and Shiga toxigenic (STEC) *E. coli.* The major exotoxins produced by the three pathotypes responsible for the enteric infections include heat-labile and heat-stable enterotoxins (mainly by ETEC), enteroaggregative heat-stable toxins (EASTs; by ETEC and EPEC), Shiga toxins (mainly by STEC), and hemolysins (mainly by EPEC and STEC). The STEC pathotype produces a subtype of Shiga toxin (Stx), called Stx2e, which causes edema disease in weaned piglets [3]. In addition, there are hybrid pathotypes, which can produce a combination of toxins [3]. Also, certain serogroups and serotypes of *E. coli* within each pathotype, based on O, H, and F antigens, are more prevalent than others in causing the diseases [4,5]. The early detection, diagnosis, treatment, and prevention of *E. coli* infections are critically important to reduce their economic impact [3,6].

Although *E. coli* is a commensal in the gut of pigs, certain serotypes and strains possess specific virulence genes to initiate infection because of the influence of predisposing factors. In addition, *E. coli*, like many other bacterial species, communicate and coordinate certain biological functions, including virulence gene expressions, through cell-density-dependent quorum sensing [7]. Undoubtedly, quorum sensing to express adhesins, including pili, and exotoxins, like hemolysins and enterotoxins, play a critical role in initiating the infection.

Feed- and water-based antimicrobials and antimicrobial alternatives are commonly employed for mitigation of enteric infections in piglets. Chlortetracycline (CTC), a broad-spectrum and medically important antibiotic, is widely used for the treatment of respiratory and enteric diseases [8,9]. Chlortetracycline can be administered orally, either in feed or in water, or as an injectable. The oral route of administration is by far the most common because of ease and convenience [5]. In a nursery pig trial, the effects of chlortetracycline (CTC), with or without direct-fed microbials (DFMs), on growth performance and antimicrobial resistance (AMR) in fecal *Escherichia coli* were assessed. CTC, alone or combined with DFMs, improved pig growth but increased AMR to tetracycline and ceftiofur in *E. coli* isolates, although this resistance generally declined over time [10]. CTC supplementation was significantly associated with an increase in tetracycline resistance in a randomized controlled trial evaluating the effects of CTC and copper in weaned pigs. This finding suggests that routine use of CTC in feed may contribute to the selection and proliferation of tetracycline-resistant bacterial populations in swine production systems [11]. Because of public health concerns associated with the development of AMR, there is considerable interest and effort in identifying feeding and management practices to minimize the prevalence of AMR in bacteria. Our objectives of the study were to (1) develop and validate a multiplex PCR assay to identify major virulence genes of the enteric pathotypes of *E. coli* in swine feces, (2) assess the impact of route of administration, in feed or in water, of CTC on prevalence of the pathotypes (ETEC, EPEC, and STEC) of *E. coli* involved in enteric colibacillosis in nursery pigs, and (3) compare the phenotypic and genotypic antimicrobial susceptibilities of *E. coli* isolates to CTC.

## 2. Materials and Methods

### 2.1. Animals and Study Design

A total of 1296 piglets (21 days of age) were housed in 48 pens with 27 piglets per pen, arranged in a randomized complete block design. Each pen was randomly assigned to one of the three treatment groups: control, in-feed CTC (22 mg/kg BW), and in-water CTC (22 mg/kg BW). The study was conducted in an enclosed commercial research nursery barn. Pens were equipped with slatted flooring, six-hole stainless steel self-feeders, and pan waterers to provide ad libitum access to feed and water. Fresh fecal samples were collected randomly from 5 of 27 piglets from each pen on days 0 (pre-treatment), 14 (treatment), and 28 (post-treatment).

### 2.2. Escherichia coli Enrichment

Approximately 1 g of fecal sample was suspended in 9 mL of *Escherichia coli* (EC) broth (Becton Dickinson and Co., Sparks, MD, USA) and vortexed for 1 min; the fecal suspension was incubated at 40 °C for 6 h. After incubation, 1 mL of the enriched fecal suspension was pipetted into a 2 mL centrifuge tube and subjected to DNA extraction [9].

### 2.3. Development and Validation of an Eleven-Plex PCR to Detect Major Virulence Genes of Enteric E. coli Pathotypes

#### 2.3.1. Gene Targets

The virulence genes were selected to identify four enteric pathotypes of *E. coli*, ETEC, EPEC, STEC, and enteroaggregative *E. coli* (EAEC), in swine feces. The targets included four enterotoxin genes, *estA*, *estB*, *elt*, and *astA*, which encode for heat-stable A, heat-stable B, heat-labile, and enteroaggregative heat-stable enterotoxins, respectively; two Shiga toxins, *stx*1 and *stx*2; adhesion factor genes, *aggA*, *eae*, and *bfpA*, which encode for subunits of enteroaggregative fimbriae, intimin, and bundle-forming pili subunit A, respectively; and two hemolysin genes, *ehxA* and *hlyA*, which encode for enterohemolysin and hemolysin A. The primers for *stx*1, *stx*2, and *ehx*A were from a previous study [12]; the adhesin factor genes primers are also from other studies [13,14,15]. The primers to amplify genes *estA*, *estB*, *elt*, *astA*, and *hlyA* were designed from the analyses of all available target sequences in this study. In addition to being specific to the target genes, the primers were designed to have a similar annealing temperature (~60 °C) and to have amplicon sizes that were well separated. The information on targeted genes and primer sequences are in Table 1.

#### 2.3.2. Assay Conditions

The working concentration of the primer mix was 4.5 pM/µL of each primer. The reaction mix, with a total volume of 20 µL, consisted of 10 µL of IQ Multiplex Powermix (Bio-Rad, Hercules, CA, USA), 7 µL of nuclease-free water (Promega, Madison, WI, USA), 1 µL of primer mix, and 2 µL of DNA extracted from enriched fecal samples. The PCR running conditions for fecal samples included initial denaturation for 2 min at 94 °C, followed by 35 cycles of 15 s denaturation at 94 °C, annealing and extension at 68 °C for 80 s, and a final extension at 68 °C for 2 min. The *E. coli* strains H10407 and sPRH-20 (ETEC), 2348-69 (EPEC), 17-2 (EAEC), and ATCC 43894 (STEC) served as our reference positive controls in the PCR assay. The PCR running conditions for pure culture isolates were the same as those for fecal samples, except the number of amplification cycles was 25.

#### 2.3.3. Sensitivity of the Assay

*Escherichia coli* strains H10407, sPRH-20, 2348-69, 17-2, and ATCC 43894 were used in pure cultures and to spike fecal samples to determine the sensitivity of the assay. A single colony of each strain was grown individually in 10 mL Luria–Bertani broth (LB; Becton Dickinson, Sparks, MD, USA) for 16 h at 37 °C. Then, 100 μL of each strain was added to another 10 mL of LB broth and incubated for 4 h at 37 °C. The bacterial cell concentrations of individual strains were determined by spread-plate count. All five strains were mixed together and seven 10-fold serial dilutions, were prepared. Each dilution was subjected to DNA extraction and PCR assay. Then 1 mL of each mixed-culture dilution (10^−1^ to 10^−7^) was added to 9 mL of EC broth suspended with swine feces, which was confirmed to be negative for most of the target genes. The spiked samples were incubated for 6 h at 40 °C, and 1 mL of each swine fecal broth suspension was collected before and after 6 h enrichment, boiled for 10 min, and centrifuged at 9300× *g* for 5 min. The supernatant of boiled broth suspension of each dilution was purified with a GeneClean Turbo kit (MP Biomedicals, Solon, OH, USA). Two microliters of purified DNA was used for the PCR assay. The experiment was repeated with the different fecal samples collected.

### 2.4. Isolation of E. coli by Direct Plating of Enriched Fecal Samples

Enriched fecal samples that were positive for one or more of the targeted virulence genes (enterotoxins, Shiga toxins, intimin) were streaked with a sterile loop onto MacConkey (MAC) agar plates (Becton Dickinson and Company, Sparks, MD, USA). Additionally, samples were diluted (1 in 100 dilution) in buffered peptone water, and 100 μL of the diluted fecal suspension was spread-plated onto MAC plates. The plates were incubated at 37 °C for 18 to 24 h. After incubation, 10 presumptive *E. coli* colonies (round, smooth, and pink-colored), positive for indole production by spot indole test, were selected and streaked onto blood agar plates (Remel, Lenexa, KS, USA) and incubated at 37 °C for 18 to 24 h. The colonies from each pie of blood agar plate were confirmed as *E. coli* by a three-plex PCR assay that targeted *uidA*, *clbB,* and *ybb* genes, which encode for beta-glucoronidase, casinolytic protease B (a heat shock protein), and putative allantoin permease, respectively. The primer sequences and amplicon size are shown in Table 2. An isolate positive for any one of the three genes was considered to be *E. coli*. The colonies of ten isolates were suspended in 100 μL of distilled water, boiled for 5 min at 95 °C, and centrifuged at 9300× *g* for 5 min, and the lysate was subjected to the 11-plex PCR assay. If pooled colonies were positive for any one or more of the virulence genes, then the ten colonies were tested individually by the 11-plex PCR to identify pure cultures positive for the virulence genes. If an isolate was positive for any of the 11 genes, it was stored using cryo-protect beads (Key Scientific Products, Stamford, TX, USA) in a −80 °C freezer.

### 2.5. Subtyping of stx2e Gene in STEC Isolates

A single-plex PCR assay was used to determine whether the *stx*2 gene was of the subtype 2e, which is involved in the edema disease, in all the STEC isolates (n = 238). The primer sequences designed were forward, CCACCAGGAAGTTATATTTCCGTA, and reverse, AACTTCACCTGGGCAAAGC. The primers’ working concentrations were 10 pM/μL. The reaction consisted of 1 μL of forward primer and 1 μL of reverse primer, 10 μL of Hot StartTaq plus Master mix (Qiagen, Hilden, Germany), 6 μL of nuclease-free water (Promega Corp., Madison, WI, USA), and 2 μL of DNA template. The total reaction volume was 20 μL. The PCR running program included an initial denaturation at 94 °C for 5 min, followed by 25 cycles of denaturation at 94 °C for 30 s, annealing at 60 °C for 30 s, extension at 67 °C for 75 s, and a final step of extension at 68 °C for 7 min. The PCR product was separated, and amplicon size was determined by using a capillary electrophoresis system, QIAxcel Advanced System, with a QIAxcel DNA Screening Kit (Qiagen, Germantown, MD, USA). The amplicon size was 1031 bp.

### 2.6. Detection of Fimbrial Genes by PCR

The purified DNA from *E. coli* isolates positive for one or more of the four enterotoxin genes was used to identify the fimbrial genes. A mPCR assay was used to detect genes that encoded for F6, F18, and F41 fimbriae, which are commonly associated with ETEC pathotypes involved in enteritis or post-weaning diarrhea. The assay also targeted putative glycosyl transferases of serogroups O8/O9, which are the main serogroups carrying the F5 gene. The primer sequences designed to amplify the genes and amplicon sizes are shown in Table 2. The assay reaction mixture included a working concentration of primer mix 1 and primer mix 2 (10 pM/uL of each primer) in a 20 µL reaction mix containing 10 µL of IQ Multiplex Powermix, 6 µL of nuclease-free water, 1 µL of primer mix 1, 1 µL of primer mix 2, and 2 µL of DNA extracted from positive isolates. The PCR running conditions were an initial denaturation for 5 min at 94 °C, followed by 25 cycles of denaturation at 94 °C for 30 s, annealing at 62 °C for 30 s, extension at 68 °C for 75 s, and final extension at 68 °C for 7 min.

### 2.7. Phenotypic and Genotypic Antimicrobial Susceptibility Determinations

A subset of isolates positive for one or more virulence genes were subjected to antimicrobial susceptibility testing of CTC by microbroth dilution method to determine the minimum inhibitory concentrations (MIC) according to the Clinical Laboratory and Standards Institute (CLSI). Stock solution of CTC antibiotic was prepared by adding sterile distilled water and the final concentration was adjusted to 1000 µg/mL. Chlortetracycline was tested at the concentrations of 100, 50, 25, 12.5, 6.25, 3.125, 1.56, 0.78, 0.39, and 0.195 µg/mL. The concentration of bacterial inoculum was adjusted to 0.5 McFarland turbidity standards (Remel Company, Lenexa, KS, USA) by adding individual bacterial colonies from the blood agar plate into 10 mL of Mueller–Hinton broth (MH; Becton, Dickinson and Co., Sparks, MD, USA) and vortexing. Then, 100 µL of the MH broth containing bacterial inoculum was dispensed into 96-well microtiter plates (Corning Incorporated, Corning, NY, USA) followed by the addition of 1:100 dilution of the culture. The microtiter plates were incubated at 37 °C for 20 to 24 h and the results were recorded as either growth or no growth. A mPCR assay was employed to detect *tet*A, *tet*B, *tet*C, and *tet*D genes [16,17].

### 2.8. Statistical Analysis

The data analysis was carried out using SAS (v. 9.4; Cary, NC, USA). Pen was considered as an experimental unit. Bivariate descriptive statistics of all the virulence genes by treatment and sampling day were carried out before building a final model. The PROC MIXED procedure with RFML (residual maximum likelihood) was used to evaluate the prevalence of virulence genes, pathotypes, their association with fimbrial genes, and tetracycline resistance genes. The final model included the fixed effects of treatment with sampling day as a random effect. The outcome variables, modeled using a binary outcome, consisted of the number of positive samples within each pen divided by the total number of samples tested per pen. The least square means were generated using the PDIFF option with a Tukey–Kramer adjustment for multiple comparisons by treatment, sampling day, and their interaction. The MIC values were log-transformed using PROC RANK and analysis was performed on ranked values. Results were considered significant at a *p*-value of <0.05.

## 3. Results

**Figure 1:**
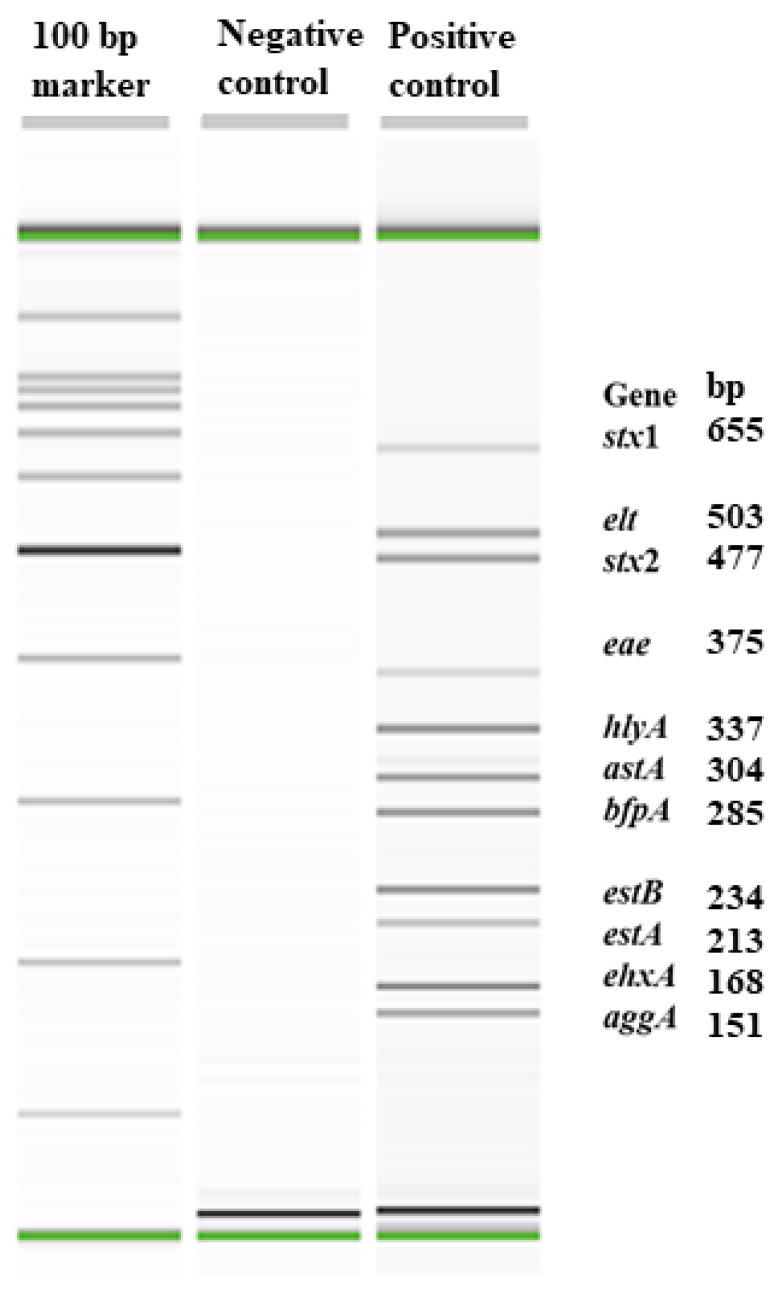
QIAxcel image of the eleven virulence genes of enteric pathotypes of *Escherichia coli* implicated in swine colibacillosis.

The optimization of the mPCR assay was achieved with DNA from two ETEC strains (H10407 and sPRH), one EPEC strain (2348-69), one EAEC strain (17-2), and one STEC strain (ATCC 43894). The amplicon sizes of the 11 virulence genes ranged from 151 to 655 bp (Figure 1). The detection limit of the assay with pooled pure cultures was 6.2×10^4^ CFU/mL and in fecal samples spiked with serially diluted, pooled pure cultures, the detection limit of the assay was 6.2×10^5^ CFU/g. A total of 360 fecal samples were analyzed, which represented 40 fecal samples from each of the three treatment groups (control, in-feed CTC, and in-water CTC) at each of the three sampling days (days 0, 14, and 28). Fecal samples were enriched in *E. coli* broth (6 h) before determining the prevalence of 11 virulence genes by mPCR assay and isolating *E. coli* positive for one or more of the virulence genes.

### 3.1. Prevalence of Virulence Genes in Fecal Samples

**Figure 2:**
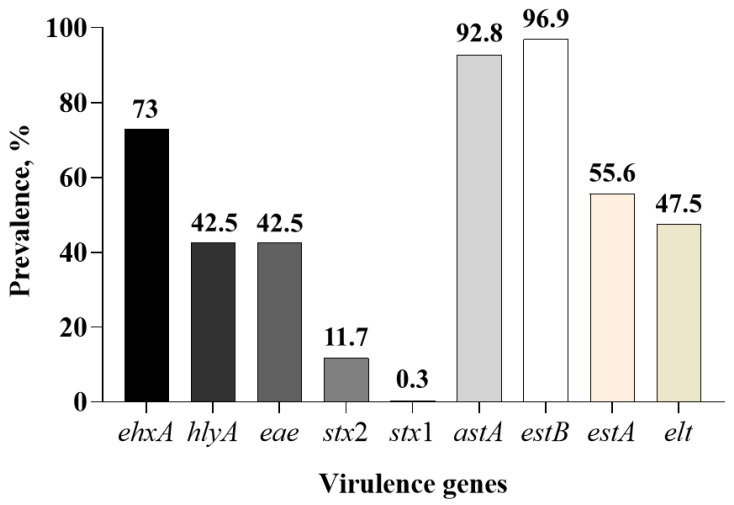
Overall sample level-based prevalence of the major virulence genes of *Escherichia coli* pathotypes involved in swine colibacillosis in fecal samples collected from piglets administered with or without in-feed or in-water chlortetracycline.

The 11 virulence genes tested were categorized into three broad groups based on their role in virulence: colonization factors (*aggA*, *bfpA*, and *eae*), enterotoxins (*elt*, *estA*, *estB*, and *astA*), and cytotoxins (*stx*1, *stx*2, *hlyA*, and *ehxA*) (Table 3). None of the 360 enriched fecal samples tested by the eleven-plex PCR assay were positive for the *aggA* or *bfpA* gene, which encode for the subunit of the enteroaggregative adherence fimbria AAF/1 and bundle-forming pilus protein, respectively. Only 4 fecal samples were negative for all 11 genes and the remaining 356 out of 360 (98.8%) samples were positive for one or more of the nine virulence genes. Overall, the prevalence of the *estB* gene (96.9%; 349/360) that encodes for the heat-stable enterotoxin B and *astA* (92.8%; 334/360) that encodes for the enteroaggregative heat-stable enterotoxin (EAST) was higher compared to the other toxin genes across all treatment groups and sampling days (Figure 2).

The prevalence of the heat-stable enterotoxin A gene, *estA*, was low (2.5%) in day 0 samples and increased to 84.2% and 80% in fecal samples collected on days 14 and 28, respectively (Table 3). Interestingly, the heat-labile enterotoxin gene, *elt*, was present in fecal samples of all three treatment groups collected on day 14 and was absent in fecal samples collected on days 0 and 28. Both *stx*1 and *stx*2 were absent in the fecal samples collected on days 0 and 14. Only one fecal sample in day 28 in the in-feed CTC group was positive for the Shiga toxin 1 gene (*stx*1). *stx*2 was prevalent in piglets of all three treatment groups and the total prevalence of *stx*2 in day 28 fecal samples was 35% (Table 3). Between the two hemolysin genes, *ehxA*, which encodes for enterohemolysin, was more prevalent (73%) than the *hlyA* gene (42.5%).

The pen-based prevalence of the nine virulence genes is shown in Table 4. In-feed or in-water CTC administration had no effect on the prevalence of the virulence genes. With the exception of *stx*1, the sampling day had significant effects (*p* < 0.05) on the prevalence of the other eight genes. However, there was no significant treatment by sampling day interaction (Table 4). The pen-level prevalence of *estB* and *astA* was 100% across all three sampling days and treatment groups. Similarly, treatment phase (day 14) and post-treatment phase (day 28) had 100% pen prevalence for *ehxA*, *estA*, and *hlyA* virulence genes. The prevalence of genes, except for *estB* and *astA*, was less than 100% in the pretreatment phase of the study (day 0). The *stx*1 gene was detected in only one pen in the in-feed CTC group. The *stx*2 gene was present in all the three treatment groups in day 28 samples, and the pen-based prevalence ranged from 37.5 to 62.5%.

### 3.2. Isolation of E. coli Positive for One or More of the Nine Virulence Genes

**Figure 3:**
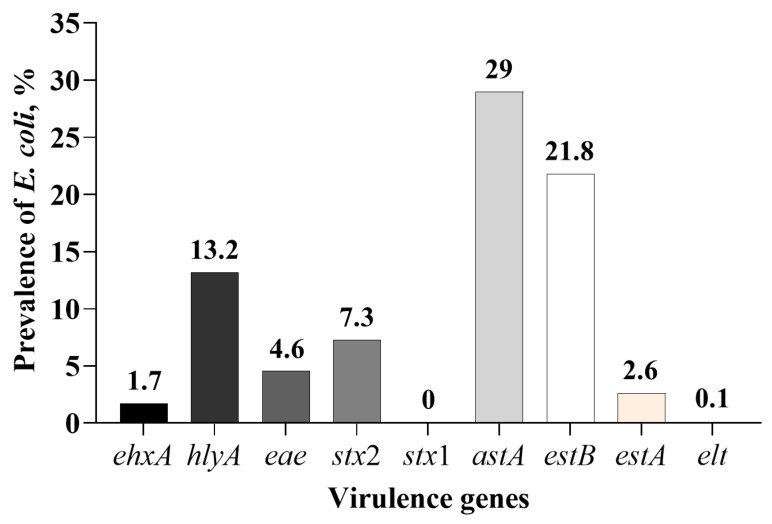
Overall sample level-based prevalence of *Escherichia coli* isolates positive for one or more of the virulence genes involved in swine colibacillosis in fecal samples collected from piglets administered with or without in-feed or in-water chlortetracycline.

A total of 3263 *E. coli* isolates positive for one or more of the nine virulence genes were obtained from fecal samples collected from piglets in the three treatment groups at the three sampling days (Table 5). Of those, 1923 isolates (58.9%) were positive for more than one gene. *E. coli* isolates positive for the two enterotoxin genes, *astA* (947/3263; 29%) and *estB* (714/3263; 21.8%), were higher than the other two enterotoxin genes, *elt* and *estA* (Figure 3). Only a small number of isolates were positive for the *estA* gene (84/3263; 2.6%). Only two isolates were positive for the *elt* enterotoxin gene. None of the isolates were positive for the *stx*1 gene. Shiga toxigenic *E. coli* were isolated from samples collected on day 28, the total number of isolates obtained was 238 out of 3263 isolates tested (7.3%), and all the isolates were positive for the *stx*2 gene only. Only a small proportion of the isolates were positive for the intimin gene, *eae* (4.6%), and none of them were positive for the *stx*2 gene. Between the two hemolysin genes tested, more isolates were positive for the *hlyA* (13.2%) than the *ehxA* (1.7%) gene.

*E. coli* isolates positive for one or more of the nine virulence genes based on pen-level prevalence are shown in Table 6. There were no treatment effects or treatment by sampling day interaction in the number of *E. coli* isolates positive for one or more of the nine virulence genes. However, except for *elt* and *estA* genes, the isolates positive for the other six genes were affected by sampling day. Between the two major enterotoxin-positive *E. coli* isolates, *astA-*positive isolates were higher in samples on day 28 than day 0 or 14, and in contrast, *estB-*positive isolates were higher in fecal samples collected on days 0 and 14 than on day 28 (Table 5 and Table 6). Shiga toxin 2-positive *E. coli* were isolated only in day 28 fecal samples.

### 3.3. Prevalence of E. coli Pathotypes

Based on the virulence genes, *E. coli* isolates were grouped into the following three pathotypes: *E. coli* positive for one or more of the four enterotoxin genes (*elt*, *estA*, *estB,* and *astA*) and negative for *eae* as enterotoxigenic *E. coli* (ETEC); *E. coli* positive for *eae* and one or more of the four enterotoxin genes and negative for Shiga toxin gene (*stx*1 or *stx*2) as atypical enteropathogenic *E. coli* (aEPEC); and *E. coli* isolates positive for Shiga toxin gene (*stx*1 or *stx*2) as Shiga toxin-producing *E. coli* (STEC) (Table 7).

**Figure 4:**
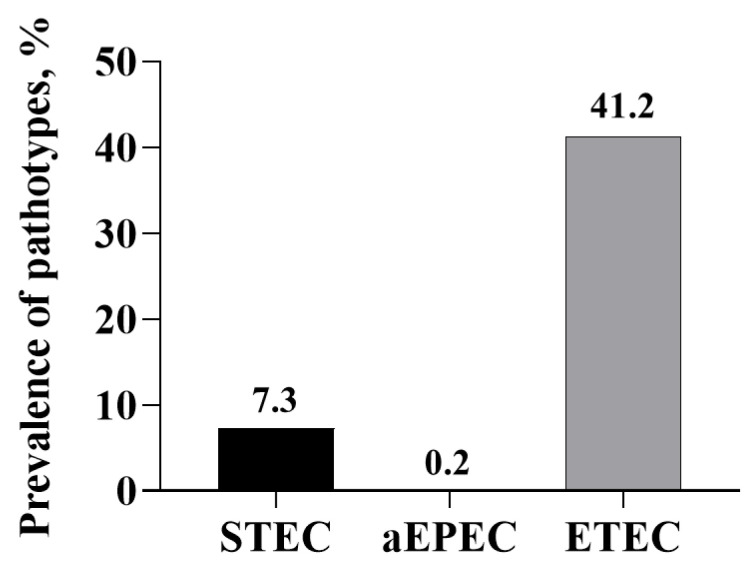
Overall sample level-based prevalence of *Escherichia coli* pathotypes involved in swine colibacillosis in fecal samples collected from piglets administered with or without in-feed or in-water chlortetracycline.

**Figure 5:**
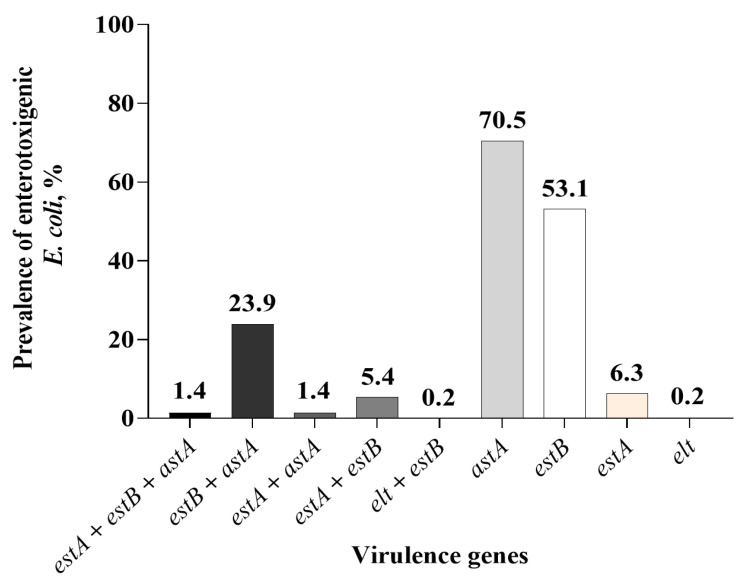
Overall sample level-based prevalence of enterotoxigenic *Escherichia coli* in fecal samples collected from piglets administered with or without in-feed or in-water chlortetracycline.

Because of the total absence of *bfpA* and *aggA*, none of the pathotypes belonged to enteropathogenic (EPEC) or enteroaggregative *E. coli*. Also, none of the isolates were of the hybrid pathotype. The ETEC pathotype was the most dominant of the three pathotypes detected (1344/3263; 41.2%; Figure 4). The ETEC pathotype, containing the *astA* gene, was the most dominant (70.5% of the ETEC isolates), followed by *estB* (53.1%; Figure 5). None of the isolates contained all four enterotoxin genes, and 19 (1.4%) contained the three enterotoxin genes (*estA*, *estB,* and *astA*). Among the ETEC containing two enterotoxin genes, more isolates contained *estB* and *astA* genes (23.9% of ETEC isolates). The number of ETEC isolates obtained increased with the sampling day. Only a small number of isolates were characterized as aEPEC (5/3263; 0.2%), and of the 5, 2 were positive for *estB*, 1 was positive for *astA* and 2 were positive for both *estB* and *astA* genes (Table 7). The STEC pathotypes were only present in day 28 samples, and all of them contained the *stx*2 gene and none were positive for *stx*1. Based on the single-plex PCR assay, all *stx*2-positive isolates contained the subtype *stx*2e. The pen level-based prevalence of the three pathotypes is shown in Table 8. There was no treatment effect nor treatment by sampling time interaction.

### 3.4. Prevalence of Fimbrial Genes

The ETEC strains (n = 1344) isolated across all treatment groups and sampling days were subjected to the mPCR assay targeting F4, F5, F6, F18, and F41 fimbriae genes (Table 9). None of the isolates was positive for F5, F6, and F41 genes. Only 11 strains were positive for fimbrial genes; 9 were positive for F4 and 2 were positive for F18. The nine F4-positive strains harbored *estA* and *estB* genes and two F18-positive strains possessed *estB* and *elt* genes.

### 3.5. Antimicrobial Susceptibility Testing

*E. coli* isolates positive for either two or more virulence genes (n = 165) were randomly selected from the three treatment groups across the three sampling days for antimicrobial susceptibility testing of CTC by microbroth dilution method. A strain with an MIC of ≥16 µg/mL was considered resistant. All the strains tested were resistant to CTC with MIC values of 50 µg/mL (CI = 37.5 to 62.5), 50 µg/mL (CI = 44.7 to 60.5), and 62.5 µg/mL (CI = 50 to 75) in the control, in-feed CTC, and in-water CTC groups, respectively (Table 10).

### 3.6. Prevalence of Tetracycline Resistance Genes

*E. coli* isolates (n = 165) used for antimicrobial susceptibility testing were further tested by PCR for the detection of *tetA*, *tetB*, *tetC*, and *tetD* genes (Table 10). None of the isolates tested positive for the *tetC* gene. *tetA* was the most predominant gene detected (151/165; 91.5%), followed by *tetB* with 16.9% prevalence (28/165), and the least dominant was *tetD* (12.7%: 21/165).

## 4. Discussion

*Escherichia coli* that causes enteric infections in animals and humans belong to six pathotypes, but four pathotypes, enterotoxigenic, enteropathogenic, Shigatoxigenic and enteroaggregative *E. coli,* are considered more common than the other two, enteroinvasive and diffusely adherent *E. coli* [18]. In order to determine the prevalence of *E. coli* pathotypes in healthy weaned piglets, a multiplex PCR assay targeting 11 major genes associated with the four pathotypes was designed and validated. Besides genes that encode for the four enterotoxin genes (heat-labile, heat-stable A, heat-stable B, and enteroaggregative heat-stable), two Shiga toxins (1 and 2), and two hemolysins (hemolysin A and enterohemolysin), *eae*, which encodes for intimin, a non-fimbrial adhesin, *bfpA*, which encodes for the type IV bundle-forming pili [19,20], and *aggA*, which encodes for fimbrial subunit of aggregative adherence fimbriae 1(AAF-1) [21], were included. The presence of *bfpA* and *aggA* in *E. coli* strains is indicative of EPEC and EAEC pathotypes, respectively. The two hemolysin genes were included because previous studies have shown an association between hemolytic activities and other virulence factors [22,23]. The *hlyA* gene encodes for alpha-hemolysin, a pore-forming cytotoxin belonging to the RTX family of toxins, and is cytotoxic to a variety of cells besides erythrocytes [24]. Although it is predominantly detected in extraintestinal pathogenic *E. coli*, it is also a major marker of swine ETEC strains, particularly those involved in post-weaning diarrhea, and is considered to enhance virulence and colonization [25]. *ehxA*, which encodes for enterohemolysin, is plasmid-borne and is widely distributed in STEC strains [26]. The presence of *ehxA* correlates with that of the Shiga toxin; therefore, it has been suggested as an epidemiological marker for STEC strains [27].

A number of PCR assays, either single-, two-, or multiplex, targeting a variety of virulence genes (toxins, fimbriae, and non-fimbrial adhesins) associated with swine pathogenic *E. coli,* have been reported [28,29,30,31,32,33,34,35]. Many of the assays have included the four enterotoxin genes and the five fimbrial genes (F4, F5, F6, F18, and F41) associated with ETEC and EPEC pathotypes. The novelty of our mPCR assay was its inclusion of genes that would facilitate identification of the four major pathotypes involved in enteric infections.

None of the fecal samples (n = 360) collected from weaned piglets and the *E. coli* isolates obtained in pure cultures (n = 3263) in the control or CTC-administered group were positive for either *bfpA* or *aggA*, which are characteristic of EPEC and EAEC, respectively. Strains of EPEC carrying the *bfpA* gene, which is located on a plasmid called the EPEC adherence factor plasmid, are described as typical EPEC because of their characteristic adherence to enterocytes, called local adherence, in which bacterial cells form clusters, called microcolonies [20]. In contrast, strains of EPEC that do not carry *bfpA* but carry the *eae* gene are described as atypical EPEC (aEPEC) [36]. In this study, only a small number of *E. coli* isolates were identified as aEPEC. The *aggA* is one of four genes (*aggA* to *aggD*) in a cluster that encodes for type I aggregative adherence fimbriae (AAF/1) required for the phenotypic expression of an aggregative adhesion pattern called ‘stacked brick’ adherence [37,38]. The EAEC is typically a human diarrheagenic *E. coli* recognized for causing persistent diarrhea in children and traveler’s diarrhea and diarrhea in immunocompromised adults [39,40]. Although the pathotype is widely prevalent in water and human food sources, it is not a pathotype generally associated with animals [41]. In a comparative genetic characterization of *E. coli* strains isolated from non-clinical samples (feces from healthy humans, companion animals, and swine) and confirmed as EAEC based on phenotypic adherence pattern, none were positive for the *aggA* gene [41]. In a comparative genomic analysis of 127 *E. coli* strains isolated from domestic animals, including swine, it was reported that 66.9% of isolates belonged to the EAEC pathotype based on a number of virulence genes (*csgA*, *aggR*, *fimA*, *astA,* etc.). Despite the prevalence of EAEC by genomic analysis, there has been no report of isolation of the EAEC pathotype from healthy or diarrheic swine [42]. However, the enterotoxin gene, *astA*, which codes for enteroaggregative heat-stable toxins has been reported in a number of studies that have characterized virulence genes in *E. coli* associated with enteric colibacillosis [22,43,44,45,46,47]. The *astA* gene of swine isolates has more than 98% homology with the nucleotide sequence of the corresponding gene from human isolates [48]. The *astA* gene is more prevalent in atypical EPEC than in typical EPEC strains implicated in diarrhea [49]. The *astA* was the second most prevalent virulence gene detected in feces at the sample level (92.8%). However, among the isolated *E. coli* strains, *astA* was the dominant enterotoxin gene (29%), followed by the *estB* gene (21.8%), and among the 947 isolates positive for the *astA* gene, 625 (65.6%) isolates were negative for the other three enterotoxin genes.

In this study, 41.2% (1351) of isolated *E. coli* strains were identified as ETEC (positive for one more of the four enterotoxin genes). The predominant enterotoxin genes in *E. coli* isolates were *estB* and *astA*, which agrees with a report of virotypes reported in weaned piglets with diarrhea or edema disease [33,34,50,51,52,53]. Only a small number of ETEC isolates (n = 9) carried fimbrial genes, *faeG* (F4; n = 9) and *fedA* (F18; n = 2). The nine F4-positive ETEC isolates carried *estA* and *estB* genes and the two F18-positive ETEC isolates carried *elt* and *estB* genes. The low prevalence of fimbrial genes in *E. coli* isolates is likely because the piglets were healthy, and none had diarrhea or edema disease. Also, it is possible that *E. coli* isolates had non-fimbrial adhesion factors, such as adhesin involved in diffuse adherence (AIDA) and porcine attaching effacing factor (*paa*) [51,53,54]. The prevalence of the *aidA* gene has been shown to be positively associated with the presence of *astA* in *E. coli* isolates in pigs with post-weaning diarrhea or edema disease [55]. In the study that reported *E. coli* pathotypes in piglets in South Korea, the predominant virotype was *estB + astA + aida* (10.4% of the ETEC isolates). The *paa*, which encodes for porcine attaching and effacing-associated protein, was first identified in EPEC isolates from swine, and is identical to the Paa protein described in *E. coli* O157:H7. The inactivation of *paa* leads to a loss of attaching and effacing activity [56].

It is interesting that Shiga toxin genes were not detected in fecal samples and no Shiga toxin-positive *E. coli* were isolated until day 28 samples. Except for one fecal sample from the in-feed CTC group, all the positive fecal samples and the isolates obtained were positive for the Shiga toxin 2 gene. Many of the published mPCR assays have targeted a subtype of the Shiga toxin 2 gene, *stx*2e, which targets endothelial cells of blood vessels, resulting in edema at specific locations [57]. The primers designed for Shiga toxin 1 and 2 genes were nonspecific to any particular subtype [58]. The intent was also to identify STEC that could also be of food safety importance [59]. Therefore, a single-plex PCR assay targeting the *stx*2e subtype was designed to test the stx2-postive *E. coli* isolates obtained (238/3263 isolates) and all the isolates were positive for the *stx*2e subtype. The absence of *stx*2e in the first 14 days of the study period is surprising because edema is typically observed in the first two weeks of the post-weaning period [60]. Normally, newborn piglets pick up the STEC strains from the sows, and it is possible that the sows in the herd were negative.

The presence of CTC resistance among these *E. coli* isolates (including the control group) suggests a high baseline prevalence of tetracycline resistance, which is attributed to the widespread use of tetracycline antibiotics in animal-based food production. The CTC supplementation was significantly associated with increased tetracycline resistance, with 99% (95% CI: 98–100%) of isolates exhibiting resistance compared to 95% (95% CI: 94–97%) in the control group [11]. This indicates that therapeutic use of CTC may further amplify existing resistance levels in fecal *E. coli* populations. Another study conducted at a U.S. research farm with no antimicrobial exposure for over five years found that *E. coli* isolates from weaned pigs exhibited higher levels of resistance to tetracycline, sulfisoxazole, and streptomycin compared to isolates from most older age groups. This finding suggests that age-related factors, such as immune system maturity or microbial community structure, may influence the persistence and distribution of antimicrobial resistance even in the absence of recent antimicrobial use [61]. Besides determining prevalence of virulence genes and pathotypes associated with enteric colibacillosis in healthy pigs, the study was designed to assess the impact of in-feed and in-water administration of CTC. The rationale for comparing in-feed and in-water administration is that the distribution of CTC in the gut is likely to be different between the two methods of administration. The distribution of in-water-administered CTC is likely to be more uniform compared to in-feed administration; therefore, the impact is likely to be different between the two groups. Neither in-feed or in-water administration of CTC had any impact on the prevalence of virulence genes or pathotypes, likely because all isolates of *E. coli* were resistant to CTC. The genotypic characterization revealed the high prevalence of *tet*A, followed by *tet*B and *tet*D. Even in previous studies, tetracycline resistance is the most prevalent resistant phenotype [62]. Tetracyclines are widely used because of their therapeutic benefits.

## 5. Conclusions

In conclusion, the use of 11-plex PCR for the detection of major virulence genes allowed us to determine the prevalence of virulence genes and pathotypes of *E. coli* associated with neonatal diarrhea, post-weaning diarrhea, and edema diseases in weaned piglets. The predominant pathotype detected was ETEC with *astA* and *estB* as the predominant enterotoxin genes. The second pathotype was *stx*2e-positive STEC and the prevalence of aEPEC was low (0.2%). Only a small number of ETEC isolates carried the fimbrial genes, likely because the piglets were healthy. All the *E. coli* isolates were resistant to chlortetracycline, with *tetA* being the predominant tetracycline-resistant gene. Overall, neither in-feed nor in-water CTC administration had any effects on the fecal prevalence of virulence genes and pathotypes implicated in swine colibacillosis.

## Figures and Tables

**Table 1:** Targeted virulence genes and virulence factors associated with enteric *Escherichia coli* pathotypes, primers, primer sequences, and amplicon size.

Virulence Genes	Virulence Factors	Primers	Primer Sequences (5′ to 3′)	Amplicon Size, bp	Source
*aggA*	Subunit of enteroaggregative adherence fimbria AAF/1	*aggA*-F1	CGTTACAAATGATTGTCCTGTTACTAT	151	Paddock et al., 2013 [13]
		*aggA*-R1	ACCTGTTCCCCATAACCAGAC		
*ehxA*	Enterohemolysin	*ehxA*-F	GCGAGCTAAGCAGCTTGAAT	168	Bai et al., 2010 [12]
		*ehxA*-R	CTGGAGGCTGCACTAACTCC		
*estA*	Heat-stable enterotoxin A	*estA*-F2	CATGACGGGAGGTAACATGA	213	This study
		*estA*-R2	GGATTACAACAAAGTTCACAGCA		
*estB*	Heat-stable enterotoxin B	*estB*-F2	CTTGACTCATATAAAAGCCCACTG	234	This study
		*estB*-R2	GCAGTACCATCTCTAACCCCTAAA		
*bfpA*	Bundle-forming pilus protein	*bfpA*-F2	CAGAAGTAATGAGCGCAACG	285	Shridhar et al., 2016 [15]
		*bfpA*-R2	CGTAGCCTTTCGCTGAAGTA		
*astA*	Enteroaggregative heat-stable enterotoxin (EAST)	*astA*-F	GGCTCAATGTGCTGACTGAA	304	This study
		*astA*-R	TGCCAGCTTCGGCTTATC		
*hlyA*	Hemolysin	*hlyA*-F4	ACGAAAGTACTGGGTAATGTTGG	337	This study
		*hlyA*-R4	ATGTCGTTGCAGCAGCACT		
*eae*	Intimin	*eae*-F2	TACGCGAAAGATACCGCTCT	375	Noll et al., 2015 [14]
		*eae*-R2	CATGCGGAAATAGCCGTTA		
*stx*2	Shiga toxin 2	*stx*1-F	CCATGACAACGGACAGCAGTT	477	Bai et al., 2010 [12]
		*stx*1-R	TGTCGCCAGTTATCTGACATTC		
*elt*	Heat-labile enterotoxin	*elt*-F2	TTATGATCACGCGAGAGGAA	503	This study
		*elt*-R2	TTGTGCTCAGATTCTGGGTCT		
*stx*1	Shiga toxin 1	*stx*1-F	TGTCGCATAGTGGAACCTCA	655	Bai et al., 2010 [12]
		*stx*1-R	TGCGCACTGAGAAGAAGAGA		

**Table 2:** Genes targeted for species confirmation of *E. coli* and fimbriae associated with enterotoxigenic *Escherichia coli* pathotypes, primer sequences, and amplicon sizes.

Genes Targeted	Encoded Protein	Primers	Primer Sequences	Amplicon Size, bp	Source
*clpB*	Caseinolytic protease B (A heat-shock protein)	*clpB*-F^#^	CATACGAATGCTGGATGCTG	449	This study
		*clpB*-R	TTTGAAGAACGTTTAAAAGGCG		
*uidA*	Beta-glucuronidase	*uidA*-F	ACCACGGTGATATCGTCCAC	449	This study
		*uidA*-R	TACAAGAAAGCCGGGCAAT		
*ybbW*	Putative allantoin permease	*ybbW*-F	AATCTGGCCGGGATTTTT	449	This study
		*ybbW*-R	TGGCTCCGGCAATAATACAT		
*faeG*	F4 fimbrial adhesin	F4-F	ATTTCAATGGTTCGGTCGAT	416	This study
		F4-R	CGCAGAAGTAACCCCACCT		
*fanC*	F5 fimbrial adhesin	F5-F	CAGGAAATACTGCTGCTAAAG	150	This study
		F5-R	GCTGGGCTGAATAGTTAAATGAC		
*fasA*	F6 fimbrial adhesin	F6-F	ACCAGCCAGGCAAATTTAGA	492	This study
		F6-R	TGTACCTGCTGAACGAATAGTCA		
*fedA*	F18 fimbrial adhesin	F18-F	CAGCAAGGGGATGTTAAATTC	218	This study
		F18-R	AACTGCCCGCTCCAAGTTA		
*f41*	F41 fimbrial adhesin	F41-F	TGATTGGACGGAAGGTCAAC	561	This study
		F41-R	CCTGGCATTAACTTTTCTACATAACC		
_	Putative glycosyltransferases	O8/O9-F	GTCTTCATCCGGGACATAGC	735	This study
		O8/O9-R	CGTGAAATCGAAGAGCTGAA		

**Table 3:** Sample level-based prevalence of the major virulence genes of *Escherichia coli* pathotypes involved in swine colibacillosis in fecal samples collected from piglets administered with or without in-feed or in-water chlortetracycline (CTC).

Virulence Factor	Day 0				Day 14				Day 28			
	Control (n = 40)	In-Feed CTC (n = 40)	In-Water CTC (n = 40)	Total, n = 120 (%)	Control (n = 40)	In-Feed CTC (n = 40)	In-Water CTC (n = 40)	Total, n = 120 (%)	Control (n = 40)	In-Feed CTC (n = 40)	In-Water CTC (n = 40)	Total, n = 120 (%)
*aggA*	0	0	0		0	0	0		0	0	0	0
*bfpA*	0	0	0		0	0	0		0	0	0	0
*eae*	5	3	2	10 (8.3%)	31	29	35	95 (79.2%)	15	15	18	48 (40%)
*elt*	0	0	0		15	23	19	57 (47.5%)	0	0	0	0
*estA*	2	1	0	3 (2.5%)	36	28	37	101 (84.2%)	33	32	31	96 (80%)
*estB*	38	37	36	111 (92.5%)	40	40	40	120 (100%)	39	40	39	118 (98.3%)
*astA*	39	40	39	118 (98.3%)	33	38	33	104 (86.7%)	37	38	37	112 (93.3%)
*stx1*	0	0	0	0	0	0	0	0	0	1	0	1 (0.83%)
*stx2*	0	0	0	0	0	0	0	0	13	16	13	42 (35%)
*hlyA*	10	9	9	28 (23.3%)	31	29	35	95 (79.2%)	15	15	18	48 (40%)
*ehxA*	14	15	9	38 (31.7%)	40	40	40	120 (100%)	33	34	37	104 (86.7%)

Sample-level prevalence calculated as the number of samples positive for the gene out of the total number of samples collected within each treatment group.

**Table 4:** Pen level-based prevalence of the major virulence genes of *Escherichia coli* pathotypes involved in swine colibacillosis in fecal samples collected from piglets administered with or without in-feed or in-water chlortetracycline (CTC).

Virulence Factor	Day 0			Day 14			Day 28			Trt	Day	Trt × Day
	Control (n = 8)	In-Feed CTC (n = 8)	In-Water CTC (n = 8)	Control (n = 8)	In-Feed CTC (n = 8)	In-Water CTC (n = 8)	Control (n = 8)	In-Feed CTC (n = 8)	In-Water CTC (n = 8)			
**Colonization factor**												
*eae*	4 (50%)	2 (25%)	2 (25%)	8 (100%)	8 (100%)	8 (100%)	6 (75%)	6 (75%)	6 (75%)	0.658	<0.001	0.769
**Enterotoxins**												
*elt*	0	0	0	5 (62.5%)	7 (87.5%)	7 (87.5%)	0	0	0	0.522	<0.001	0.623
*estA*	1 (12.5%)	1 (12.5%)	0	8 (100%)	7 (87.5%)	8 (100%)	8 (100%)	8 (100%)	8 (100%)	0.301	<0.001	0.311
*estB*	8 (100%)	8 (100%)	8 (100%)	8 (100%)	8 (100%)	8 (100%)	8 (100%)	8 (100%)	8 (100%)	-	-	-
*astA*	8 (100%)	8 (100%)	8 (100%)	8 (100%)	8 (100%)	8 (100%)	8 (100%)	8 (100%)	8 (100%)	-	-	-
**Cytotoxins**												
*stx*1	0	0	0	0	0	0	0	1 (12.5)	0	0.376	0.377	0.418
*stx*2	0	0	0	0	0	0	4 (50%)	5 (62.5%)	3 (37.5%)	0.926	<0.001	0.989
*hlyA*	6 (75%)	5 (62.5%)	8 (100%)	8 (100%)	8 (100%)	8 (100%)	8 (100%)	7 (87.5%)	8 (100%)	0.707	<0.001	0.982
*ehxA*	5 (62.5%)	8 (100%)	6 (75%)	8 (100%)	8 (100%)	8 (100%)	8 (100%)	8 (100%)	8 (100%)	0.920	<0.001	0.337

Pen-level prevalence calculated as the number of pens that had at least one positive sample for the gene divided by the total number of pens sampled per treatment group.

**Table 5:** Sample level-based prevalence of *Escherichia coli* positive for the virulence genes associated with enteric pathotypes involved in swine colibacillosis in fecal samples from piglets administered with or without in-feed or in-water chlortetracycline (CTC).

Virulence Factor	Day 0				Day 14				Day 28			
	Control (n = 40)	In-Feed CTC (n = 40)	In-Water CTC (n = 40)	Total, (n = 120)	Control (n = 40)	In-Feed CTC (n = 40)	In-Water CTC (n = 40)	Total (n = 120)	Control (n = 40)	In-Feed CTC (n = 40)	In-Water CTC (n = 40)	Total (n = 120)
No. of isolates	310	280	279	869	394	400	400	1194	400	400	400	1200
*eae*	8 (2.6) ^a^	21 (7.5)	41 (14.7)	70 (8.1)	21 (5.3)	23 (5.8)	22 (5.5)	66 (5.5)	8 (2)	2 (0.5)	5 (1.3)	15 (1.3)
*elt*	0	0	0	0	2 (0.5)	0	0	2 (0.2)	0	0	0	0
*estA*	7 (2.3)	0	0	7 (0.8)	21 (5.3)	12 (3)	16 (4)	49 (4.1)	3 (0.8)	12 (3)	13 (3.3)	28 (2.3)
*estB*	99 (31.9)	112 (40)	83 (29.7)	294 (33.8)	78 (19.8)	154 (38.5)	145 (36.3)	377 (31.6)	2 (0.5)	16 (4)	25 (6.3)	43 (6)
*astA*	98 (32)	102 (36.4)	92 (33)	292 (33.6)	31 (7.9)	40 (10)	34 (8.5)	105 (8.8)	163 (40.8)	209 (52.3)	178 (44.5)	550 (45.8)
*stx*1	0	0	0	0	0	0	0	0	0	0	0	0
*stx*2	0	0	0	0	0	0	0	0	90 (22.5)	75 (18.7)	73 (18.2)	238 (19.8)
*hlyA*	15 (4.8)	9 (3.2)	9 (3.2)	33 (3.8)	73 (18.5)	29 (7.3)	35 (8.8)	137 (11.5)	97 (24.3)	87 (21.8)	76 (19)	260 (21.6)
*ehxA*	12 (3.9)	0	0	12 (1.4)	9 (2.3)	15 (3.8)	10 (2.5)	34 (2.8)	5 (1.3)	1 (0.3)	4 (1)	10 (0.8)

^a^ Numbers in parentheses are percentage of isolates positive for the virulence gene out of the total isolates obtained in each treatment group.

**Table 6:** Pen level-based prevalence of *Escherichia coli* positive for the virulence genes associated with enteric pathotypes involved in swine colibacillosis in fecal samples from piglets received CTC via feed, water, or no treatment.

Virulence Genes	Day 0			Day 14			Day 28			*p*-value		
	Control	In-Feed CTC	In- Water CTC	Control	In-Feed CTC	In- Water CTC	Control	In-Feed CTC	In- Water CTC	Trt	Day	Trt × Day
**Colonization factor**												
*eae*	2 (25%)	3 (37.5%)	4 (50%)	6 (75%)	6 (75%)	6 (75%)	3 (37.5%)	2 (25%)	2 (25%)	0.257	0.030	0.102
**Enterotoxins**												
*elt*	0	0	0	1 (12.5%)	0	0	0	0	0	0.377	0.377	0.418
*estA*	1 (12.5%)	0	0	5 (62.5%)	4 (50%)	6 (75%)	3 (37.5%)	3 (37.5%)	2 (25%)	0.881	0.054	0.525
*estB*	6 (75%)	7 (87.5%)	6 (75%)	6 (75%)	7 (87.5%)	7 (87.5%)	2 (25%)	3 (37.5%)	4 (50%)	0.268	< 0.001	0.829
*astA*	6 (75%)	7 (87.5%)	6 (75%)	6 (75%)	6 (75%)	5(62.5%)	7 (87.5%)	7 (87.5%)	7 (87.5%)	0.737	< 0.001	0.976
**Cytotoxins**												
*stx*1	0	0	0	0	0	0	0	0	0	0	0	0
*stx*2	0	0	0	0	0	0	3 (37.5%)	3 (37.5%)	3 (37.5%)	0.964	0.001	0.997
*hlyA*	2 (25%)	3 (37.5%)	2 (25%)	8 (100%)	4 (50%)	6 (75%)	6 (75%)	5 (62.5%)	6 (75%)	0.576	0.016	0.963
*ehxA*	2 (25%)	0	0	4 (50%)	6 (75%)	4 (50%)	2 (25%)	1 (12.5%)	2 (25%)	0.387	0.069	0.256

Pen-level prevalence calculated as the number of pens that had at least one positive sample for the gene divided by the total number of pens sampled per treatment group (n = 8).

**Table 7:** Sample level-based prevalence of the *Escherichia coli* pathotypes involved in swine colibacillosis in fecal samples collected from piglets receiving CTC via feed or water or no treatment.

Pathotypes	Day 0				Day 14				Day 28			
	Control (n = 40)	In-Feed CTC (n = 40)	In-Water CTC (n = 40)	Total (n = 120)	Control (n = 40)	In-Feed CTC (n = 40)	In-Water CTC (n = 40)	Total (n = 120)	Control (n = 40)	In-Feed CTC (n = 40)	In-Water CTC (n = 40)	Total (n = 120)
No. of isolates	310	280	279	869	394	400	400	1194	400	400	400	1200
ETEC ^a^	107	111	93	311	104	190	152	446	168	220	199	587
Heat-labile (*elt)*	0	0	0	0	2	0	0	2	0	0	0	0
Heat-stable A (*estA)*	7	0	0	7	21	12	16	49	3	12	13	28
Heat-stable B (*estB)*	99	111	83	294	78	154	145	377	2	16	25	43
Enteroaggregative heat-stable (*astA*)	98	102	92	292	31	40	34	105	163	209	178	550
*elt + estB*	0	0	0	0	2	0	0	2	0	0	0	0
*estA + estB*	7	0	0	7	21	11	16	48	0	8	10	18
*estA + astA*	0	0	0	0	2	1	1	4	0	8	7	15
*estB + astA*	90	102	82	274	4	5	23	32	0	8	7	15
*estA + estB + astA*	0	0	0	0	2	1	1	4	0	8	7	15
aEPEC ^b^	0	0	0	0	1	1	2	4	0	1	0	1
STEC ^c^	0	0	0	0	0	0	0	0	90	75	73	238

^a^ ETEC pathotype: Isolates positive for one or more of the four enterotoxin genes (*elt*, *estA*, *estB*, and *astA*). ^b^ Atypical EPEC pathotype: Isolates positive for *eae* gene, negative for Shiga toxin genes (*stx*1 or *stx*2), and positive for one or more of the four enterotoxin genes (*elt*, *estA*, *estB*, and *astA*). ^c^ STEC pathotype: Isolates positive for one or both of the Shiga toxin genes (*stx*1 and *stx*2).

**Table 8:** Pen level-based prevalence of *Escherichia coli* pathotypes implicated in swine colibacillosis in fecal samples collected from piglets administered with or without in-feed or in-water chlortetracycline (CTC) and enriched in *E. coli* broth.

Pathotype	Day 0			Day 14			Day 28			Trt	Day	Trt × Day
	Control (n = 8)	In-Feed CTC (n = 8)	In-Water CTC (n = 8)	Control (n = 8)	In-Feed CTC (n = 8)	In-Water CTC (n = 8)	Control (n = 8)	In-Feed CTC (n = 8)	In-Water CTC (n = 8)			
Enterotoxigenic *E. coli* ^a^	6 (75%)	7 (87.5%)	6 (75%)	7 (87.5%)	7 (87.5%)	7 (87.5%)	7 (87.5%)	7 (87.5%)	7 (87.5%)	0.929	0.640	0.977
Atypical Enteropathogenic *E. coli* ^b^	0	0	0	1 (12.5%)	1 (12.5%)	2 (25%)	0	1 (12.5%)	0	0.815	0.004	0.895
Shigatoxigenic *E. coli* ^c^	0	0	0	0	0	0	3 (37.5%)	3 (37.5%)	3 (37.5%)	1.000	<0.001	1.000

^a^ ETEC pathotype: Isolates positive for one or more of the four enterotoxin genes (*elt*, *estA*, *estB*, and *astA*). ^b^ Atypical EPEC pathotype: Isolates positive for *eae* gene, negative for Shiga toxin genes (*stx*1 or *stx*2) and positive for one or more of the four enterotoxin genes (*elt*, *estA*, *estB*, and *astA*). ^c^ STEC pathotype: Isolates positive for one or both of the Shiga toxin genes (*stx*1 and *stx*2).

**Table 9:** Prevalence of fimbrial genes in enterotoxigenic *Escherichia coli* isolated from fecal samples collected from piglets received CTC via feed or water or no treatment and enriched in *E. coli* broth.

Fimbriae Genes	Day 0				Day 14				Day 28				Overall Total, n = 1344
	Control (n = 107)	In-Feed CTC (n = 111)	In-Water CTC (n = 93)	Total, n = 311	Control (n = 104)	In-Feed CTC (n = 190)	In-Water CTC (n = 152)	Total, n = 446	Control (n = 168)	In-Feed CTC (n = 220)	In-Water CTC (n = 199)	Total, n = 587	
F4-*faeG*	7	0	0	7	1	1	0	2	0	0	0	0	9 *
F5-*fanC*	0	0	0	0	0	0	0	0	0	0	0	0	0
F6-*fasA*	0	0	0	0	0	0	0	0	0	0	0	0	0
F18-*fedA*	0	0	0	0	2	0	0	2	0	0	0	0	2 **
F41-*f41*	0	0	0	0	0	0	0	0	0	0	0	0	0

* Nine F4-positive isolates were positive for virulence genes *estA* and *estB*. ** Two F18-positive isolates were also positive for virulence genes *estB* and *elt.*

**Table 10:** Minimum inhibitory concentrations (MICs) of chlortetracycline (CTC) for *Escherichia coli* (n = 165) isolated from fecal samples collected from piglets administered with or without in-feed or in-water CTC.

Treatment Groups	Pathotypes	No. of Isolates	Chlortetracycline, µg/mL		Tetracycline Resistance Genes (%, Proportion)		
			MIC	95% Confidence Interval	*tetA*	*tetB*	*tetD*
Control	ETEC ^a^	35	54.2	[44.9–63.6]	31 (88.5%)	5 (14.3%)	4 (11.4%)
	STEC ^b^	3	45.8	[27.9–63.7]	0	3 (100%)	0
	Others ^c^	14	62.2	[43.3–81.2]	14 (100%)	1 (7.1%)	4 (28.5%)
	Total	52	50	[37.5–62.5]	45 (86.5%)	9 (17.3%)	8 (15.4%)
In-feed CTC	ETEC ^a^	41	53.9	[46.6–61.2]	40 (97.5%)	5 (12.2%)	3 (7.3%)
	STEC ^b^	3	43.7	[28.2–59.3]	0	3 (100%)	0
	Others ^c^	13	75.5	[58.3–92.8]	13 (100%)	1 (7.7%)	2 (15.4%)
	Total	57	50	[44.7–60.5]	53 (93%)	9 (15.8%)	5 (8.7%)
In-water CTC	ETEC ^a^	36	58.8	[49.6–68.0]	35 (97.2%)	5 (13.8%)	5 (13.8%)
	STEC ^b^	3	45.8	[36.8–54.7]	2 (66.6%)	3 (100%)	0
	Others ^c^	17	74.4	[58.2–90.6]	16 (94.1%)	2 (11.7%)	3 (17.6%)
	Total	56	62.5	[50–75]	53 (94.6%)	10 (17.8%)	8 (14.3%)

MIC breakpoint for CTC resistance is ≥16 µg/mL. ^a^ ETEC pathotype: Isolates positive for one or more of the four enterotoxin genes (*elt*, *estA*, *estB*, and *astA*). ^b^ STEC pathotype: Isolates positive for one or both of the Shiga toxin genes (*stx*1 and *stx*2). ^c^ Others: Isolates positive for both ETEC and aEPEC pathotypes (4) and remaining 40 isolates, which are positive for the *eae* gene.

## Data Availability

The original contributions presented in this study are included in the article. Further inquiries can be directed to the corresponding author.

## Abbreviations

The following abbreviations are used in this manuscript:

*E. coli*	*Escherichia coli*
ETEC	Enterotoxigenic *Escherichia coli*
EPEC	Enteropathogenic *Escherichia coli*
STEC	Shigatoxigenic *Escherichia coli*
aEPEC	Atypical enteropathogenic *Escherichia coli*
CTC	Chlortetracycline
PCR	Polymerase chain reaction
*tet*	Tetracycline
AMR	Antimicrobial resistance
MAC	MacConkey
mPCR	Multiplex polymerase chain reaction
MIC	Minimum inhibitory concentration
CLSI	Clinical Laboratory and Standards Institute
PWD	Post-weaning diarrhea
*elt*	Heat-labile enterotoxin
*estA*	Heat-stable enterotoxin A
*estB*	Heat-stable enterotoxin B
*astA*	Enteroaggregative heat-stable enterotoxin (EAST)
*stx 1*	Shiga toxin 1
*stx 2*	Shiga toxin 2
*eae*	Intimin
*hlyA*	Hemolysin
*ehxA*	Enterohemolysin
*aggA*	Subunit of enteroaggregative adherence fimbria AAF/1
*bfpA*	Bundle-forming pilus protein
